# Effect of fluid balance situation within 7 days and early fluid intake after admission to the intensive care unit on in-hospital mortality and 1-year mortality in patients with cardiac arrest: a retrospective study from the MIMIC IV database

**DOI:** 10.3389/fcvm.2025.1519306

**Published:** 2025-11-11

**Authors:** Lei Zhang, Chang Liu, Xin Sui, Jian Zhang, Wenjia Xu, Yufei Sun, Chengke Yin, Fei Han

**Affiliations:** Department of Anesthesiology, Harbin Medical University Cancer Hospital, Harbin, Heilongjiang, China

**Keywords:** cardiac arrest, fluid resuscitation, fluid balance, intravenous fluid, medical information mart for intensive care IV

## Abstract

**Background:**

The objective of this study was to assess the associations between the mean daily fluid balance within 7 days and fluid intake within 24 h after admission to intensive care unit (ICU) and mortality for hospitalization and 1-year in cardiac arrest (CA) patients.

**Methods:**

Patients who experienced CA were enrolled from the Medical Information Mart for Intensive Care Database. CA patients were divided into <14, 14–37, 38–79 and >79 ml.kg^−1^ groups according to the interquartile range of the mean daily fluid balance. In addition, patients were divided into low (<147 ml.kg^−1^) fluid intake group and high (≥147 ml.kg^−1^) fluid intake group according to the median fluid intake within 24 h after admission to the ICU. Multivariate logistic regression models were constructed to determine the independent risk factors for in-hospital mortality and 1-year mortality.

**Results:**

The in-hospital mortality in the 38–79 ml.kg^−1^ and the >79 ml.kg^−1^ groups were higher than in the 14–37 ml.kg^−1^ and the <14 ml.kg^−1^ groups (*P* < 0.05). The 1-year mortality in the 38–79 ml.kg^−1^ and the >79 ml.kg^−1^ groups were higher than in the <14 ml.kg^−1^ group (*P* < 0.05). The 38–79 ml.kg^−1^ and >79 ml.kg^−1^ groups were associated with increased risk of in-hospital mortality [odds ratio (OR) 2.300, 95% confidence interval (CI) 1.381–3.831; *P* = 0.001; OR 2.691, 95% CI 1.515–4.779; *P* = 0.001] and 1-year mortality (OR 2.131, 95% CI 1.308–3.470; *P* = 0.002; OR 2.141, 95% CI 1.237–3.703; *P* = 0.006). The in-hospital mortality in the high-fluid intake group was higher than in the low-fluid intake group (*P* < 0.05). The 1-year mortality was not significantly different between the two groups (*P* = 0.055). A high fluid intake was not associated with an increased risk of in-hospital mortality (OR 0.841, 95% CI 0.587–1.204; *P* = 0.344).

**Conclusion:**

Mean daily fluid balance ≥38 ml.kg^−1^ within 7 days after admission to the ICU was associated with increased in-hospital mortality and 1-year mortality in cardiac arrest patients. Fluid intake ≥147 ml.kg^−1^ within 24 h after admission to the ICU was not associated with increased in-hospital mortality in cardiac arrest patients.

## Introduction

Cardiac arrest (CA) is a major global health problem associated with high early and long-term mortality (1). Survival has been increasing with improved resuscitation therapies, but the overall survival after CA is still less than 30% (2). Multiple factors are associated with the prognosis of CA patients, including the place where CA occurs, the presence of witnesses, the initial rhythm, and the duration of cardiopulmonary resuscitation (CPR) (3). Current post-CA treatment includes multiple interventions, including chest compressions, ventilation, and early defibrillation as the foundation of CA treatment, as well as the use of medications, IVF, and targeted temperature management, among other technological tools (4–6). Ischemia‒reperfusion injury due to CA has deleterious effects on body systems. This usually leads to the development of post-CA syndrome, which mainly consists of intravascular volume reduction, vasodilation, endothelial injury and microcirculatory abnormalities, resulting in hemodynamic instability and thus requiring fluid therapy (7, 8). Intravenous fluid (IVF) may increase preload and improve cardiac output and blood pressure after the return of spontaneous circulation (ROSC) (9). However, excessive fluid intake during resuscitation may exacerbate cardiac load and promote fluid transfer to the interstitial space, leading to edema and organ dysfunction (10).

A large cross-sectional analytical study showed that IVF (vs. no IVF) could increase prehospital survival in out-of-hospital CA patients; however, this strategy did not improve long-term mortality (11). Fahad Gul observed no significant difference in 30-day mortality after admission to intensive care unit (ICU) between the restricted (<30 ml.kg^−1^) IVF group and liberal (>30 ml.kg^−1^) IVF group in CA patients (12). A retrospective study showed that greater fluid volume (4–5 L infused in the first 24 h) reduced the incidence of acute kidney injury in patients with cardiogenic shock after CA (13). Therefore, the association between IVF and mortality in CA patients is unclear. The objective of this retrospective study was to analyze the effect of the mean daily fluid balance within 7 days and fluid intake within 24 h after admission to the ICU on in-hospital mortality and 1-year mortality in CA patients.

## Methods

### Data source

The data used in this retrospective observational study were obtained from the Medical Information Mart for Intensive Care (MIMIC-IV) database (version 1.0). The database was constructed and maintained by the Harvard Medical School and the Computational Physiology Laboratory at Massachusetts Institute of Technology and was approved by the Beth Israel Deaconess Medical Center and the Massachusetts Institute of Technology Institutional Review Board. The cohort included all the medical records of 256,878 patients admitted to the ICU or emergency department between 2008 and 2019 at Beth Israel Deaconess Medical Center. The researchers were free of access to the database after passing the online training provided by the National Institutes of Health (Record ID: 48236799). The MIMIC-IV database has strict privacy protection processing, and all patient-protected information was data-processed and hidden; therefore, the consent of patients was waived.

### Participants

Adult CA patients in the MIMIC-IV database with International Classification of Diseases-9 (ICD-9) diagnosis codes 4275 (cardiac arrest) were selected. Exclusion criteria: (1) Patients who did not experience CA or who suffered CA due to hemorrhage, pregnancy, or surgery; (2) Age <18 years; (3) Less than 24 h in the ICU; (4) IVF and weight data collected within 24 h after admission to the ICU were lacking.

### Data extraction

The Structured Query Language (SQL) tool with PgAdmin4 was used to extract the following data from the MIMIC-IV database for the study (if there were multiple records, the average value was taken). The demographic characteristics and comorbidities included age, sex, ethnicity, weight, duration of ICU admission, hypertension, coronary heart disease (CHD), diabetes, acute heart failure (AHF), congestive heart failure (CHF), and chronic obstructive pulmonary disease (COPD). The laboratory parameters included potassium, sodium, calcium, blood urea nitrogen (BUN), blood creatinine, blood lactate, pH and blood glucose. The vital signs and other indicators included systolic blood pressure (SBP), diastolic blood pressure (DBP), mean arterial pressure (MAP), heart rate (HR), respiratory rate (RR), arterial oxyhemoglobin saturation (SpO_2_). Treatment included the use of vasopressors (epinephrine, norepinephrine, vasopressin, dopamine, and dobutamine), diuretics (furosemide, torsemide, and chlorthalidone), continuous renal replacement therapy (CRRT) and mechanical ventilation. The severity scores included the sequential organ failure assessment (SOFA), simplified acute physiology score II (SAPS II) and Glasgow coma scale (GCS) score. All laboratory data were extracted from the data generated within the first 24 h after the patients entered the ICU. Daily fluid intake and output after the patients entered the ICU (for 7 consecutive days) were recorded. Daily fluid balance was calculated from total daily fluid intake minus total fluid output. The endpoint of our study were in-hospital mortality and 1-year mortality. The in-hospital mortality was defined as survival status at the hospital discharge, it was determined by the time of death as recorded in the database is less than or equal to the time of discharge. The 1-year mortality was defined as survival status at 1-year after discharge, it was determined by the time of death recorded in the database is less than or equal to the time of 1-year after discharge.

First, we investigated the impact of the mean daily fluid balance within 7 days after admission to the ICU on the mortality rate of patients with CA. We collected data on the mean daily fluid intake of all patients. The interquartile range can describe the distribution of data in more detail by dividing the data into four parts, which helps to observe the differences in the characteristics of patients within different intervals. Patients were divided into four groups according to the interquartile range of the mean daily fluid balance: <14, 14–37, 38–79, and >79 ml.kg^−1^. In addition, we studied the impact of fluid intake within 24 h after admission to the ICU on the mortality rate of patients with CA. We determined the median value of the fluid intake within 24 h, which is suitable for quickly distinguishing the middle position of the data and the distribution on both sides. The patients were divided into low (<147 ml.kg^−1^) and high (≥147 ml.kg^−1^) fluid intake groups according to the median fluid intake.

### Outcomes

The primary outcome was to investigate the associations between mean daily fluid balance within 7 days after admission to the ICU and in-hospital mortality and 1-year mortality of CA patients. The secondary outcome was to investigate the associations between fluid intake within 24 h after admission to the ICU and in-hospital mortality and 1-year mortality of CA patients.

### Statistical analysis

Categorical variables are expressed as frequencies and percentages and were compared by chi-square tests or Fisher's exact tests. Normally distributed continuous variables are expressed as the means ± standard deviations (SDs), and differences between groups were tested using Student's *t* test and one-way ANOVA. Nonnormally distributed continuous variables are expressed as medians with interquartile ranges (IQRs) and were compared by the nonparametric rank sum test (Mann‒Whitney *U* test and Kruskal‒Wallis test). Multiple interpolation was used to estimate and fill in the missing data. The proportion of missing values in the collected data is no more than 20%. Since more than 10% of the lactate concentration and pH data were missing in the model, they were transformed into dummy variables to avoid possible bias from filling in the missing values directly using the mean method. Variables with less than 5% missing data were replaced with the mean value. All the time records from the extracted data were converted to a common time format.

Multivariate logistic regression models were constructed to determine the independent risk factors for in-hospital mortality and 1-year mortality. Variables with *P* < 0.05 in the univariate analysis were included in the multivariate logistic regression model. All the data were processed and statistically analyzed using STATA/MP17 software (College Station, Texas) and IBM SPSS Statistics software (version 26.0, IBM Corp, Armonk, NY, USA). *P* < 0.05 indicates statistical significance.

## Results

**Figure 1:**
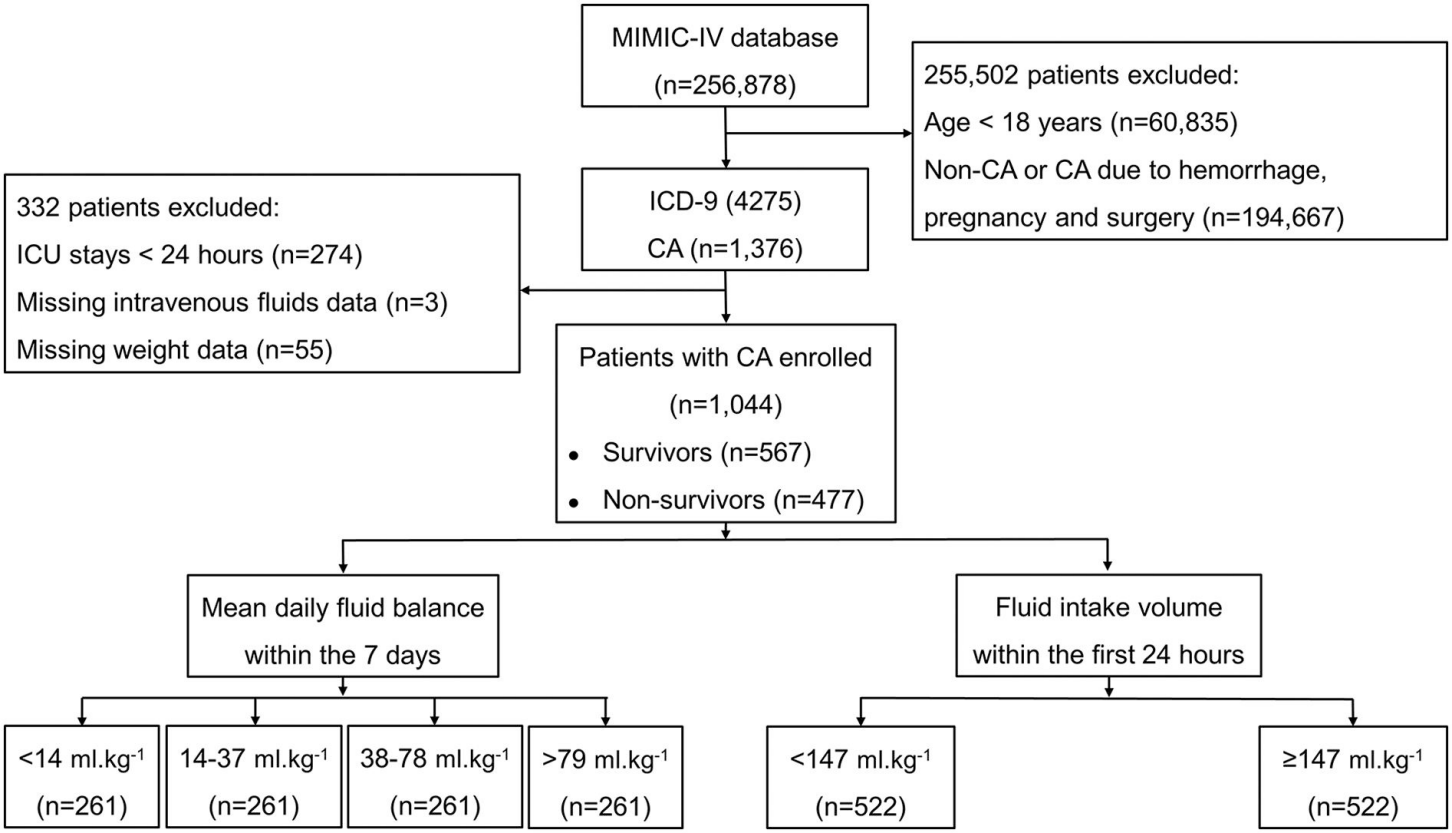
Flow chart of the distribution of patients in the MIMIC-IV database, inclusion and exclusion criteria of patients. CA, cardiac arrest.

A total of 256,878 patients admitted to the ICU between 2008 and 2019 were extracted from the MIMIC-IV database (Figure 1). Of these, 60,835 individuals aged <18 years and 194,667 individuals who did not experience CA or who suffered CA due to hemorrhage, pregnancy or surgery were excluded. A total of 1,376 patients were diagnosed with CA; 274 were admitted to the ICU less than 24 h, 3 lacked infusion data, and 55 had missing weight data. A total of 1,044 patients were ultimately enrolled in this study, including 567 in-hospital survivors and 477 in-hospital nonsurvivors. There were 261 patients in each of the four groups: <14, 14–37, 38–79 and >79 ml.kg^−1^. Of the 1,044 patients, the low-fluid intake group and the high-fluid intake group each consisted of 522 patients. The detailed missing data are shown in Supplementary Table S1.

### Characteristics of in-hospital survivors and in-hospital nonsurvivors

The characteristics of the in-hospital survivors and in-hospital nonsurvivors are shown in Table 1. The median age of the study population was 67.8 (57–79.9) years, of which 60.7% were male. Compared with the survivors, the nonsurvivors had lower body weight (*P* = 0.001) and SBP (*P* = 0.027); faster HR (*P* < 0.001) and RR (*P* < 0.001); higher SOFA score (*P* < 0.001) and SAPS II score (*P* < 0.001); lower GCS score (*P* < 0.001); greater use of vasopressors (73.2% vs. 55.6%, *P* < 0.001); greater use of mechanical ventilation (82.4% vs. 75.8%, *P* = 0.01); lower use of diuretics (58.7% vs. 74.1%, *P* < 0.001); greater use of CRRT (15.1% vs. 9.3%, *P* = 0.004); and shorter ICU stays (*P* = 0.027). The percentages of patients with AHF (17% vs. 22.9%, *P* = 0.017), CHF (36.1% vs. 43.2%, *P* = 0.019), hypertension (39.4% vs. 44.6%, *P* = 0.09), CHD (40.0% vs. 48.3%, *P* < 0.001), and COPD (2.3% vs. 4.6%, *P* = 0.047) were lower among the nonsurvivors than among the survivors. Creatinine, BUN, glucose and sodium levels were greater among the nonsurvivors than among the survivors. The percentage of patients with a pH < 7.35 was greater (54.3% vs. 39.5%, *P* < 0.001), and the percentage of patients with a lactate concentration ≥2.6 mmol/L was greater (56.8% vs. 32.3%, *P* < 0.001) among nonsurvivors. Compared with survivors, nonsurvivors had greater fluid intake within 24 h after admission to the ICU (164.8 [78.4, 271.2] ml.kg^−1^ vs. 128.7 [58.7, 220.2] ml.kg^−1^, *P* < 0.001) and greater mean daily fluid balance (50.9 [23.1, 91.5] ml.kg^−1^ vs. 26.9 [8.4, 60.5] ml.kg^−1^, *P* < 0.001). The other variables were not significantly different.

**Table 1:** Characteristics of in-hospital survivors and in-hospital nonsurvivors.

Variables	Total (*n* = 1,044)	Survivors (*n* = 567)	Nonsurvivors (*n* = 477)	*P* value
Age (years)	67.8 (57.0, 79.9)	65.4 (56.4, 77.6)	69.5 (58.3, 80.6)	0.002
Gender—*n* (%)				0.001
Female	410 (39.3%)	197 (34.7%)	213 (44.7%)	
Male	634 (60.7%)	370 (65.3%)	264 (55.3%)	
Ethnicity—*n* (%)				<0.001
White	649 (62.2%)	375 (66.1%)	274 (57.4%)	
Black	109 (10.4%)	57 (10.1%)	52 (10.9%)	
Other	286 (27.4%)	135 (23.8%)	151 (31.7%)	
Weight (kg)	80.3 (67.6, 96.0)	81.0 (68.5, 96.4)	80.0 (67.0, 95.2)	0.001
MAP (mmHg)	76.7 ± 10.7	77.2 ± 9.4	76.1 ± 12.1	0.116
SBP (mmHg)	114.5 ± 15.3	115.4 ± 13.9	113.3 ± 16.7	0.027
DBP (mmHg)	61.4 ± 11.2	61.8 ± 10.2	61.0 ± 12.3	0.23
HR (bpm)	84.8 ± 17.2	82.6 ± 16.2	87.5 ± 18.0	<0.001
RR (bpm)	20.3 ± 4.2	19.6 ± 4.0	21.1 ± 4.2	<0.001
SpO_2_ (%)	97.8 (96.3, 99.1)	97.9 (96.6, 99.1)	97.6 (96.0, 99.2)	0.07
Scoring systems				
SOFA	8.0 (5.0, 12.0)	7.0 (4.0, 11.0)	10.0 (6.0, 13.0)	<0.001
SAPS Ⅱ	45.0 (35.0, 57.8)	40.0 (31.5, 52.0)	50.0 (41.0, 63.0)	<0.001
GCS	12.0 (4.0, 15.0)	13.0 (8.0, 15.0)	10.0 (3.0, 15.0)	<0.001
Treatment				
Vasopressor—*n* (%)	664 (63.6%)	315 (55.6%)	349 (73.2%)	<0.001
Ventilation—*n* (%)	823 (78.8%)	430 (75.8%)	393 (82.4%)	0.01
Diuretics—*n* (%)	700 (67.0%)	420 (74.1%)	280 (58.7%)	<0.001
CRRT—*n* (%)	125 (12.0%)	53 (9.3%)	72 (15.1%)	0.004
Comorbidities				
AHF—*n* (%)	211 (20.2%)	130 (22.9%)	81 (17.0%)	0.017
CHF—*n* (%)	417 (39.9%)	245 (43.2%)	172 (36.1%)	0.019
Hypertension—*n* (%)	441 (42.2%)	253 (44.6%)	188 (39.4%)	0.09
Diabetes mellitus—*n* (%)	826 (79.1%)	441 (77.8%)	385 (80.7%)	0.245
CHD—*n* (%)	436 (41.8%)	274 (48.3%)	162 (40.0%)	<0.001
COPD—*n* (%)	37 (3.5%)	26 (4.6%)	11 (2.3%)	0.047
Laboratory tests				
Creatinine (mg/dl)	1.3 (0.9, 2.1)	1.1 (0.8, 1.8)	1.5 (0.9, 2.5)	<0.001
BUN (mg/dl)	25.3 (17.3, 40.2)	22.0 (15.7, 35)	28.3 (19.3, 46.8)	<0.001
Glucose (mg/dl)	156.2 (127.4, 199.4)	148.7 (124.5, 186.6)	165.6 (133, 217.8)	<0.001
Sodium (mmol/L)	138.5 ± 4.7	138.1 ± 4.3	138.8 ± 5.2	0.02
Potassium (mmol/L)	4.3 ± 0.6	4.3 ± 0.5	4.3 ± 0.6	0.781
Calcium (mg/dl)	8.3 ± 0.8	8.4 ± 0.8	8.3 ± 0.9	0.031
pH—*n* (%)				<0.001
<7.35	483 (46.3%)	224 (39.5%)	259 (54.3%)	
≥7.35	439 (42.0%)	267 (47.1%)	172 (36.1%)	
No test	122 (11.7%)	76 (13.4%)	46 (9.6%)	
Lactate (mmol/L)—*n* (%)				<0.001
<2.6	445 (42.6%)	292 (51.5%)	153 (32.1%)	
≥2.6	454 (43.5%)	183 (32.3%)	271 (56.8%)	
No test	145 (13.9%)	92 (19.2%)	53 (11.1%)	
Mean daily fluid balance (ml.kg^−1^)	38.4 (13.5, 79.0)	26.9 (8.4, 60.5)	50.9 (23.1, 91.5)	<0.001
Fluid intake within the first 24 h (ml.kg^−1^)	147.0 (66.4, 243.3)	128.7 (58.7, 220.2)	164.8 (78.4, 271.2)	<0.001
Length of stay in ICU (hours)	99.1 (55.0, 202.5)	100.6 (58.0, 212.8)	93.4 (52.1, 185.3)	0.027

MAP, mean arterial pressure; SBP, systolic blood pressure; DBP, diastolic blood pressure; HR, heart rate; RR, respiration rate; SpO_2_, arterial oxyhemoglobin saturation; SOFA, sequential organ failure assessment; SAPSⅡ, simplified acute physiology scores Ⅱ; GCS, glasgow coma scale; CRRT, continuous renal replacement therapy; AHF, acute heart failure; CHF, congestive heart failure; CHD, coronary heart disease; COPD, chronic obstructive pulmonary disease; BUN, blood urea nitrogen; ICU, intensive care unit.

### Fluid status of survivors and nonsurvivors within 7 days after admission to the ICU

**Figure 2:**
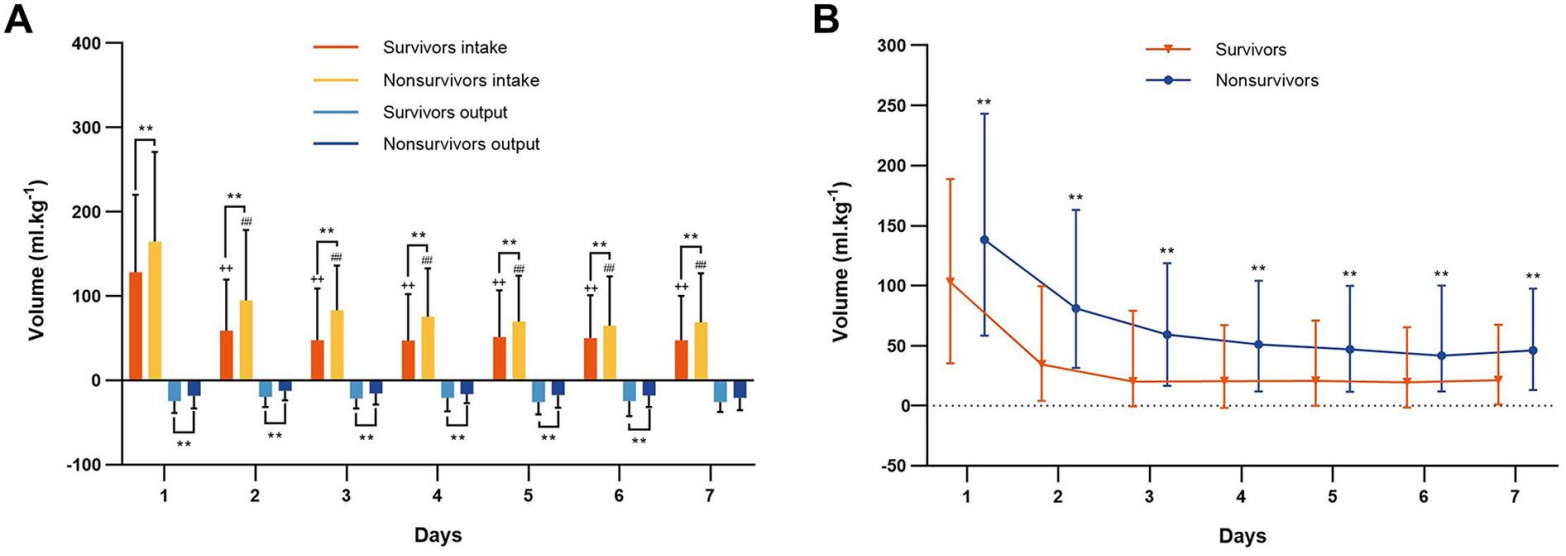
Fluid intake, fluid output and fluid balance of survivors and nonsurvivors. **(A)** Daily fluid intake and output of survivors and nonsurvivors during the 7 consecutive days after cardiac arrest. **P < 0.001 vs. survivors. ^++^*P* < 0.001, vs. the first day fluid intake of the survivors, ^##^*P* < 0.001, vs. the first day fluid intake of the nonsurvivors. **(B)** Daily fluid balance of survivors and nonsurvivors during the 7 consecutive days after cardiac arrest. **P < 0.001 vs. survivors.

**Figure 3:**
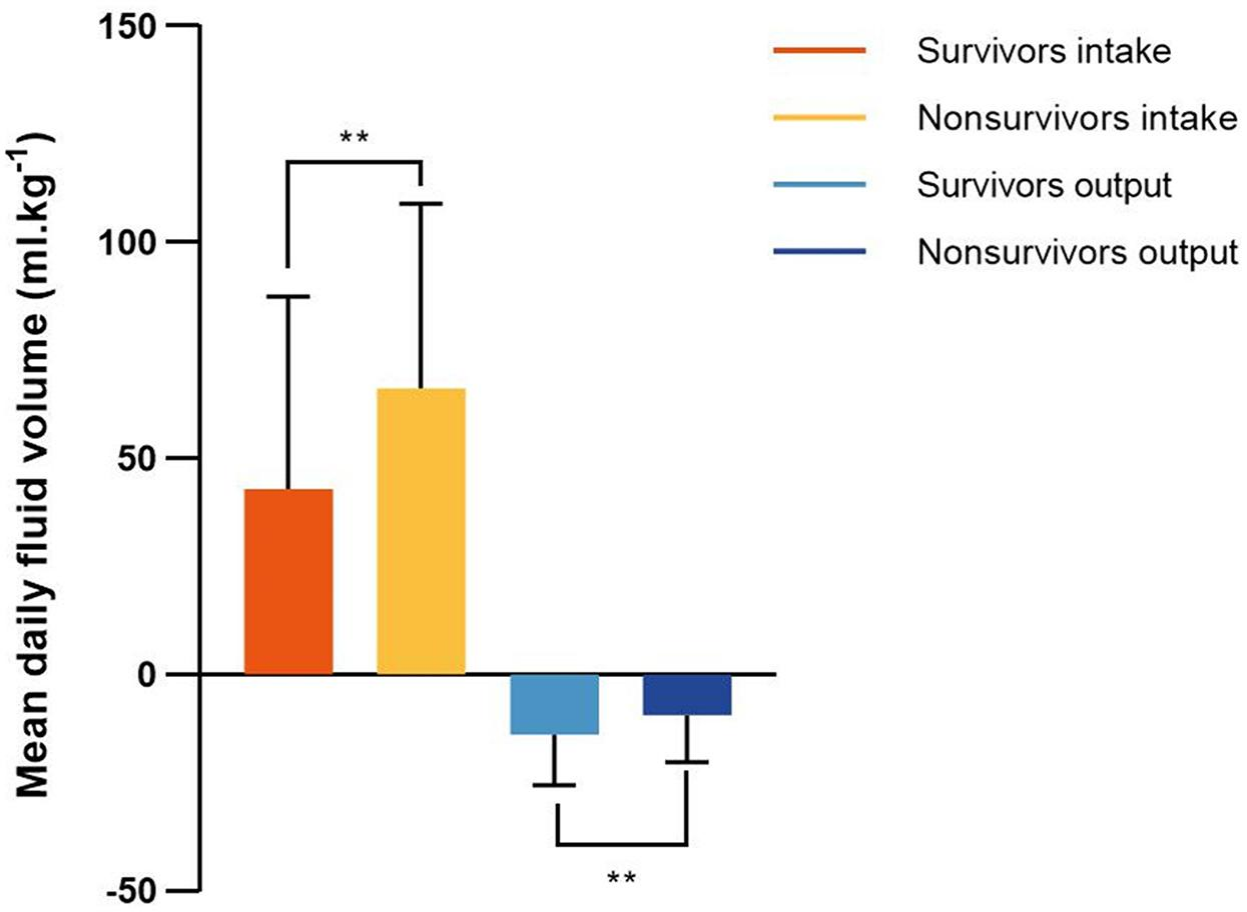
Mean fluid intake and output in survivors and nonsurvivors within 7 days after admission to the intensive care unit. Mean daily fluid intake was higher in nonsurvivors than that in survivors, and mean daily fluid output was lower in nonsurvivors than that in survivors. **P < 0.001.

**Figure 4:**
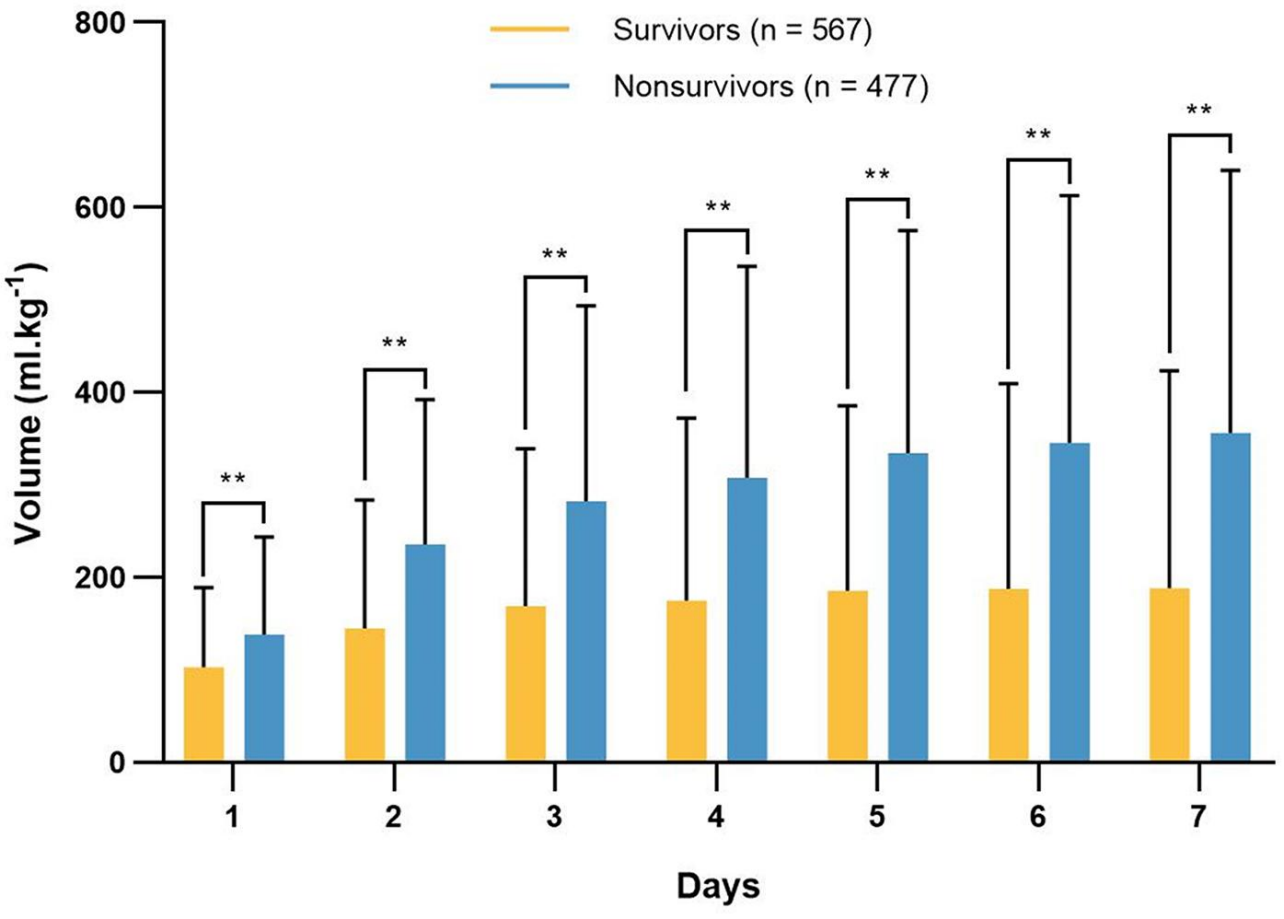
Cumulative fluid balance of survivors and nonsurvivors in each day 7 consecutive days after admission to the intensive care unit. The cumulative fluid balance increased over time, and it was higher in nonsurvivors compared with survivors. **P < 0.001.

The daily fluid intake, fluid output and fluid balance of survivors and nonsurvivors within 7 days after admission to the ICU are shown in Figure 2. Compared with survivors, daily fluid intake was greater in nonsurvivors (*P* < 0.001; Figure 2A). However, within the first 6 days, daily fluid output was lower in nonsurvivors than in survivors (*P* < 0.001), and there was no significant difference between the survivors and nonsurvivors on the 7th day (*P* = 0.31). Moreover, survivors and nonsurvivors had significantly greater fluid intake on the first day than on the other 6 days (*P* < 0.001). The fluid balance was significantly greater in nonsurvivors than in survivors within 7 days (*P* < 0.001; Figure 2B). The mean daily fluid intake was greater in nonsurvivors than in survivors (66.3 [32.1, 109] ml.kg^−1^ vs. 42.9 [17.9, 87.4] ml.kg^−1^, *P* < 0.001), and the mean daily fluid output was lower in nonsurvivors than in survivors (9.5 [3.4, 20.2] ml.kg^−1^ vs. 13.9 [6.2, 25.5] ml.kg^−1^, *P* < 0.001; Figure 3). In addition, the cumulative fluid balance increased over time in both groups and was greater in nonsurvivors than in survivors during the 7-day period (*P* < 0.001; Figure 4).

### Primary outcome

The demographic characteristics, comorbidities, severity scores and biochemical indices of the individuals in the four mean daily fluid balance groups are shown in Table 2. Patients in the >79 ml.kg^−1^ group had lower body weights and greater SBP than did those in the other three groups (*P* < 0.05). Patients in the >79 ml.kg^−1^ group had faster HR, RR and greater use of diuretics than did those in the <14 and 14–37 ml.kg^−1^ groups (*P* < 0.05). The SpO_2_ concentration in the >79 ml.kg^−1^ group was lower than that in the 14–37 and 38–79 ml.kg^−1^ groups (*P* < 0.05). Compared with those in the other three groups, patients in the >79 ml.kg^−1^ group had higher SOFA scores and SAPS II scores, lower GCS scores, greater use of vasopressors, greater mechanical ventilation ratios and greater use of CRRT (*P* < 0.05). The >79 ml.kg^−1^ group had a lower incidence of AHF (16.9% vs. 35.2%, *P* < 0.05), CHF (35.2% vs. 47.1%, *P* < 0.05) and CHD (36.4% vs. 49.8%, *P* < 0.05) than did the <14 ml.kg^−1^ group. The >79 ml.kg^−1^ group had greater creatinine levels, greater glucose levels and lower calcium levels than did the <14 ml.kg^−1^ group (*P* < 0.05). BUN levels in the 38–79 ml.kg^−1^ group were greater than those in the <14 ml.kg^−1^ group (*P* < 0.05). The percentage of patients with a pH < 7.35 was greater (62.8%) in the >79 ml.kg^−1^ group than in the other three groups (*P* < 0.05). The percentage of patients with a lactate concentration ≥2.6 mmol/L was greater (61.3%) in the >79 ml.kg^−1^ group than in the <14 and 14–37 ml.kg^−1^ groups (*P* < 0.05). The mean daily fluid balance was 118 (96.2, 158.3) ml.kg^−1^, and the fluid intake was 268 (185.6, 391.8) ml.kg^−1^ in the >79 ml.kg^−1^ group; these values were significantly greater than those in the other three groups (*P* < 0.05). The length of stay in the ICU was the longest in the >79 ml.kg^−1^ group (205.9 [109.4, 383.3], *P* < 0.05). The other variables were not significantly different.

**Table 2:** Baseline characteristics of patients in the mean daily fluid balance groups.

Variables	Mean daily fluid balance (ml.kg^−1^)				*P* value
	<14 (*n* = 261)	14–37 (*n* = 261)	38–79 (*n* = 261)	>79 (*n* = 261)	
Age (years)	67.9 (58.5, 80.4)	68.3 (56.7, 79.9)	66.2 (55.8, 77.9)	68.5 (57.8, 79.9)	0.332
Gender—*n* (%)					0.432
Female	95 (36.4%)	97 (37.2%)	109 (41.8%)	109 (41.8%)	
Male	166 (63.6%)	164 (62.8%)	152 (58.2%)	152 (58.2%)	
Ethnicity—*n* (%)					0.13
White	181 (69.3%)	160 (61.3%)	152 (58.2%)*	156 (59.8%)	
Black	25 (9.6%)	25 (9.6%)	32 (12.3%)	27 (10.3%)	
Other	55 (21.1%)	76 (29.1%)	77 (29.5%)	78 (29.9%)	
Weight (kg)	81.8 (67.5, 100.0)	83.7 (70.1, 98.0)	80 (68.4, 95.1)	78 (65.5, 90.9)*^,^**^,^***	0.001
MAP (mmHg)	76.4 ± 10.8	76.8 ± 10.7	77.9 ± 11.1	75.9 ± 10.2	0.166
SBP (mmHg)	117 ± 15.3	114.6 ± 14.9	115.3 ± 16.1	110.9 ± 14.2*^,^**^,^***	<0.001
DBP (mmHg)	61.9 ± 11.3	61.1 ± 10.9	62.4 ± 11.6	60.3 ± 11.0	0.154
HR (bpm)	81.7 ± 15.8	83.8 ± 18.4	85.9 ± 16.8*	88 ± 17.2*^,^**	<0.001
RR (bpm)	19.4 ± 3.6	20.0 ± 4.0	20.8 ± 4.4*^,^**	21.1 ± 4.5*^,^**	<0.001
SpO_2_ (%)	97.3 (95.9, 98.6)	98.1 (96.7, 99.2)*	98.1 (96.7, 99.4)*	97.7 (95.9, 99.0)**^,^***	<0.001
Scoring systems					
SOFA	4.0 (2.0, 7.0)	8.0 (5.0, 10.0)*	10.0 (7.0, 13.0)*^,^**	12.0 (9.0, 14.0)*^,^**^,^***	<0.001
SAPS Ⅱ	35.0 (26.0, 44.0)	45.0 (35.0, 57.0)*	48.0 (38.0, 62.0)*^,^**	54.0 (43.0, 66.0)*^,^**^,^***	<0.001
GCS	14.0 (13.0, 15.0)	13.0 (7.0, 14.0)*	9.0 (3.0, 14.0)*^,^**	7.0 (3.0, 12.0)*^,^**^,^***	<0.001
Treatment					
Vasopressor—*n* (%)	59 (22.6%)	154 (59.0%)*	211 (80.8%)*^,^**	240 (81.7%)*^,^**^,^***	<0.001
Ventilation—*n* (%)	122 (46.7%)	206 (78.9%)*	240 (92.0%)*^,^**	255 (97.7%)*^,^**^,^***	<0.001
Diuretics—*n* (%)	164 (62.8%)	164 (62.8%)	178 (68.2%)	194 (74.3%)*^,^**	0.014
CRRT—*n* (%)	5 (1.9%)	11 (4.2%)	25 (9.6%)*	84 (32.2%)*^,^**^,^***	<0.001
Comorbidities					
AHF—*n* (%)	76 (29.1%)	52 (19.9%)	39 (14.9%)*	44 (16.9%)*	<0.001
CHF—*n* (%)	123 (47.1%)	108 (41.4%)	94 (36.0%)	92 (35.2%)*	0.019
Hypertension—*n* (%)	110 (42.1%)	116 (44.4%)	117 (44.8%)	98 (37.5%)	0.309
Diabetes mellitus—*n* (%)	190 (72.8%)	210 (80.5%)	215 (82.4%)	211 (80.8%)	0.033
CHD—*n* (%)	130 (49.8%)	120 (46.0%)	91 (34.9%)*	95 (36.4%)*	0.001
COPD—*n* (%)	11 (4.2%)	6 (2.3%)	8 (3.1%)	12 (4.6%)	0.466
Laboratory tests					
Creatinine (mg/dl)	1.1 (0.9, 1.8)	1.2 (0.8, 2.0)	1.4 (1.0, 2.2)*	1.4 (0.9, 2.3)*^,^**	0.004
BUN (mg/dl)	22.2 (15.7, 38.4)	25.8 (15.8, 39.0)	27.9 (18.5, 42.0)*^,^**	24.8 (18.3, 42.0)	0.022
Glucose (mg/dl)	136.3 (114.0, 174.5)	160.8 (129.5, 198.1)*	161.0 (134.0, 218.8)*	161.8 (135.2, 208.1)*	<0.001
Sodium (mmol/L)	138.0 ± 4.5	138.5 ± 4.6	138.5 ± 4.8	138.8 ± 5.1	0.233
Potassium (mmol/L)	4.3 ± 0.6	4.3 ± 0.5	4.3 ± 0.6	4.3 ± 0.6	0.319
Calcium (mg/dl)	8.6 ± 0.7	8.3 ± 0.8*	8.2 ± 0.8*	8.2 ± 0.9*	<0.001
pH—*n* (%)					<0.001
<7.35	71 (27.2%)	114 (43.7%)*	134 (51.3%)*	164 (62.8%)*^,^**^,^***	
≥7.35	120 (46.0%)	117 (44.8%)	110 (42.1%)	92 (35.2%)	
No test	70 (26.8%)	30 (11.5%)*	17 (6.5%)	5 (1.9%)*	
Lactate (mmol/L)—*n* (%)					<0.001
<2.6	122 (46.7%)	128 (49.0%)	107 (41.0%)	88 (33.7%)*^,^**	
≥2.6	59 (22.6%)	97 (37.2%)*	138 (52.9%)*^,^**	160 (61.3%)*^,^**	
No test	80 (30.7%)	36 (13.8%)*	16 (6.1%)*^,^**	13 (5.0%)*^,^**	
Mean daily fluid balance (ml.kg^−1^)	4.8 (0.7, 8.8)	24.1 (18.1, 32.0)*	53.8 (45.0, 65.3)*^,^**	118.0 (96.2, 158.3)*^,^**^,^***	<0.001
Fluid intake within the first 24 h (ml.kg^−1^)	48.4 (31.8, 75.2)	125.3 (77.3, 180.5)*	205.3 (127.2, 297.0)*^,^**	268.0 (185.6, 391.8)*^,^**^,^***	<0.001
Length of stay in ICU (hours)	63.1 (39.6, 97.4)	83.5 (50.2, 142.3)*	121.2 (70.1, 216)*^,^**	205.9 (109.4, 383.3)*^,^**^,^***	<0.001

MAP, mean arterial pressure; SBP, systolic blood pressure; DBP, diastolic blood pressure; HR, heart rate; RR, respiration rate; SpO_2_, arterial oxyhemoglobin saturation; SOFA, sequential organ failure assessment; SAPSⅡ, simplified acute physiology scores Ⅱ; GCS, glasgow coma scale; CRRT, continuous renal replacement therapy; AHF, acute heart failure; CHF, congestive heart failure; CHD, coronary heart disease; COPD, chronic obstructive pulmonary disease; BUN, blood urea nitrogen; ICU, intensive care unit.

* *P* < 0.05, vs. mean daily fluid balance <14 ml.kg^−1^.

** *P* < 0.05, vs. mean daily fluid balance14–37 ml.kg^−1^.

*** *P* < 0.05, vs. mean daily fluid balance = 38–79 ml.kg^−1^.

**Figure 5:**
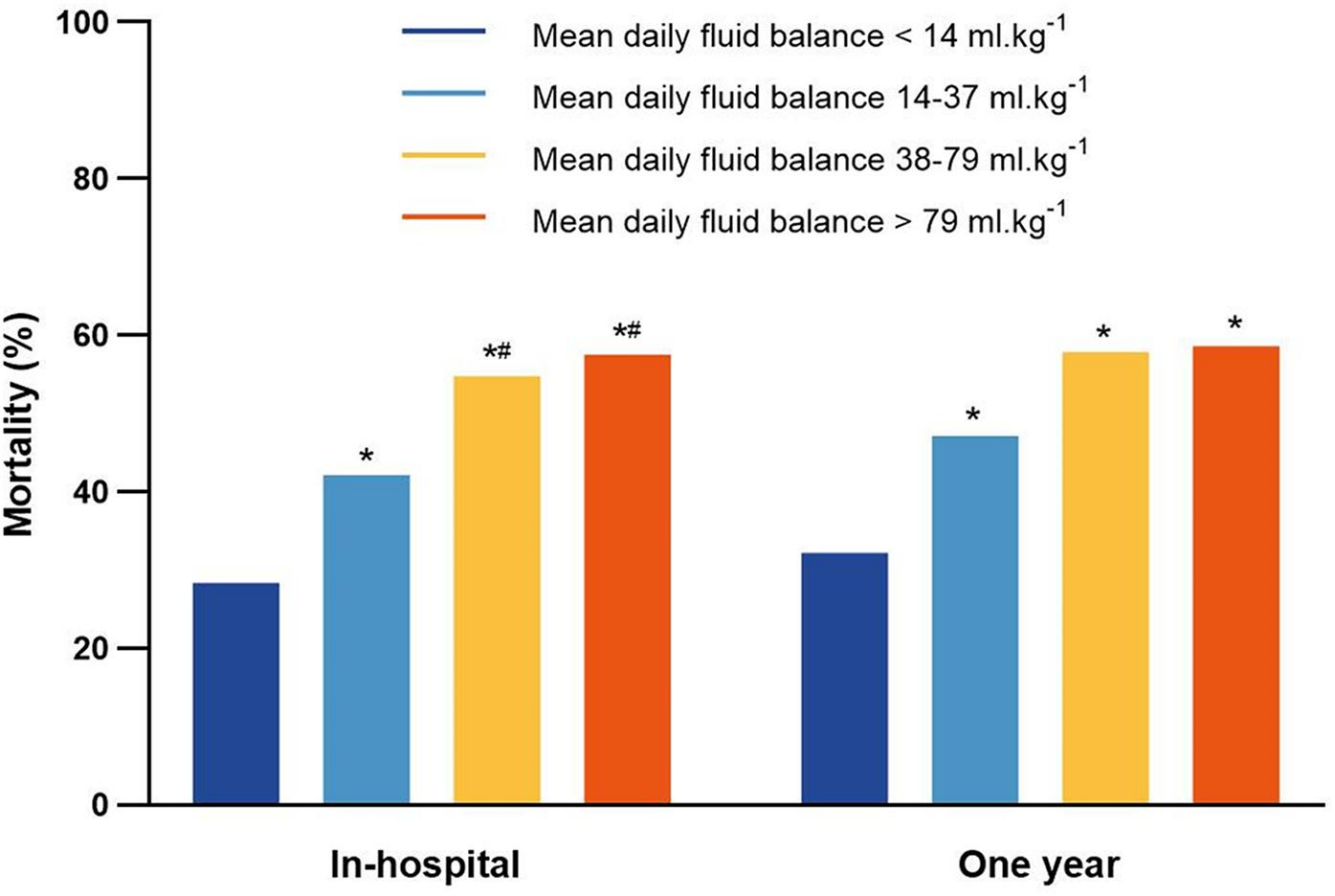
The in-hospital mortality and 1-year mortality for patients in the mean daily fluid balance groups. *P < 0.05, vs. mean daily fluid balance <14 ml.kg^−1^; ^#^*P* < 0.05, vs. mean daily fluid balance14–37 ml.kg^−1^.

The in-hospital mortality was 28.4% (74 deaths of 261 patients) in the <14 ml.kg^−1^ group, which was significantly lower than those in the other three groups (*P* < 0.05). The in-hospital mortality was 42.1% (110 deaths of 261 patients) in the 14–37 ml.kg^−1^ group, lower than 54.8% (143 deaths of 261 patients) in the 38–79 ml.kg^−1^ group and 57.5% (150 deaths of 261 patients) in the >79 ml.kg^−1^ group (*P* < 0.05). However, there was no significant difference between the 38–79 ml.kg^−1^ group and the >79 ml.kg^−1^ group (Figure 5). The 1-year mortality was the 47.1% (123 deaths of 261 patients) in the 14–37 ml.kg^−1^ group, 57.9% (151 deaths of 261 patients) in the 38–79 ml.kg^−1^ group and 58.6% (153 deaths of 261 patients) in the >79 ml.kg^−1^ group, which were significantly higher than 32.2% (84 deaths out of 261) in the <14 ml.kg^−1^ group (*P* < 0.05).

Variables (*P* < 0.05) screened by univariate logistic regression analysis for association with in-hospital mortality included age, sex, ethnicity, SBP, HR, RR, SpO_2_, SOFA, SAPS II, GCS, vasopressor use, mechanical ventilation ratios, diuretic use, CRRT, AHF, CHF, CHD, blood creatinine, BUN, glucose, sodium, calcium, pH, lactate, mean daily fluid balance and length of stay in the ICU (Supplementary Table S2). In addition, variables (*P* < 0.05) screened by univariate logistic regression analysis for association with 1-year mortality included in the multivariate logistic regression analysis (Supplementary Table S3). The associations between the mean daily fluid balance and in-hospital mortality and between the mean daily fluid balance and 1-year mortality in CA patients after adjustment for the effects of confounding factors are shown in Tables 3, 4. Compared with the <14 ml.kg^−1^ group, the 38–79 ml.kg^−1^ group and the >79 ml.kg^−1^ group were associated with greater in-hospital mortality (OR 2.300, 95% CI 1.381–3.831; *P* = 0.001; OR 2.691, 95% CI 1.515–4.779; *P* = 0.001). Similarly, compared with the <14 ml.kg^−1^ group, the 38–79 ml.kg^−1^ group and the >79 ml.kg^−1^ group were associated with greater 1-year mortality (OR 2.131, 95% CI 1.308–3.470; *P* = 0.002; OR 2.141, 95% CI 1.237–3.703; *P* = 0.006).

**Table 3:** Multivariate logistic regression analysis of in-hospital mortality.

Variables	Multivariate model^a^		
	OR	95% CI	*P* value
Age	1.021	1.009–1.033	<0.001
Male	0.706	0.518–0.962	0.027
HR	1.014	1.004–1.023	0.005
SOFA	0.930	0.873–0.991	0.026
GCS	0.881	0.884–0.919	<0.001
Diuretics	0.454	0.319–0.646	<0.001
Vasopressor	1.508	1.006–2.259	0.046
CHD	0.596	0.434–0.820	0.001
BUN	1.025	1.016–1.035	<0.001
Lactate			0.001
<2.6 mmol/L	Reference		
≥2.6 mmol/L	1.984	1.398–2.816	<0.001
No test	1.182	0.672–2.078	0.562
Mean daily fluid balance			0.003
<14 ml.kg^−1^	Reference		
14–37 ml.kg^−1^	1.459	0.928–2.293	0.101
38–79 ml.kg^−1^	2.300	1.381–3.831	0.001
>79 ml.kg^−1^	2.691	1.515–4.779	0.001
Length of stay in ICU	0.997	0.996–0.998	<0.001

OR, odd ratio; CI, confidence interval; HR, heart rate; SOFA, sequential organ failure assessment; GCS, glasgow coma scale; CHD, coronary heart disease; BUN, blood urea nitrogen, ICU, intensive care unit.

a Variables (*P* < 0.05) screened by univariate logistic regression analysis for association with in-hospital mortality included in the multivariate logistic regression analysis, including age, gender, ethnicity, SBP, HR, RR, SpO_2_, SOFA, SAPSⅡ, GCS, vasopressor use, mechanical ventilation ratios, diuretics use, CRRT, AHF, CHF, coronary heart disease, blood creatinine, BUN, glucose, sodium, calcium, pH, lactate, mean daily fluid balance and length of stay in ICU.

**Table 4:** Multivariate logistic regression analysis of 1-year mortality.

Variables	Multivariate model^a^		
	OR	95% CI	*P* value
Age	1.020	1.009–1.031	<0.001
HR	1.018	1.008–1.027	<0.001
GCS	0.900	0.863–0.937	<0.001
Diuretics	0.481	0.345–0.670	<0.001
Vasopressor	1.581	1.067–2.341	0.022
CHD	0.658	0.486–0.892	0.007
BUN	1.023	1.014–1.032	<0.001
Lactate			<0.001
<2.6 mmol/L	Reference		
≥2.6 mmol/L	1.983	1.411–2.788	<0.001
No test	1.274	0.734–2.211	0.390
Mean daily fluid balance			0.018
<14 ml.kg^−1^	Reference		
14–37 ml.kg^−1^	1.536	0.997–2.368	0.052
38–79 ml.kg^−1^	2.131	1.308–3.470	0.002
>79 ml.kg^−1^	2.141	1.237–3.703	0.006
Length of stay in ICU	0.998	0.997–0.998	<0.001

OR, odd ratio; CI, confidence interval; HR, heart rate; GCS, glasgow coma scale; CHD, coronary heart disease; BUN, blood urea nitrogen; ICU, intensive care unit.

a Variables (*P* < 0.05) screened by univariate logistic regression analysis for association with 1-year mortality included in the multivariate logistic regression analysis, including age, gender, SBP, HR, RR, SpO_2_, SOFA, SAPSⅡ, GCS, vasopressor use, mechanical ventilation ratios, diuretics use, CRRT, hypertension, coronary heart disease, blood creatinine, BUN, glucose, pH, lactate, mean daily fluid balance, length of stay in ICU.

### Secondary outcome

The characteristics of patients in the low and high fluid intake groups within the first 24 h are shown in Table 5. The fluid intake was 243.2 (191.2, 344.1) ml.kg^−1^ in the high-fluid intake group and 66.4 (40.2, 101.5) ml.kg^−1^ in the low-fluid intake group. Compared with those in the low-fluid intake group, the high-fluid intake group had longer ICU stays (118.2 [63.8, 244.2] vs. 83.6 [50.9, 183.1], *P* < 0.001), greater CRRT rates (15.7% vs. 8.2%, *P* < 0.001), greater use of vasopressors (84.3% vs. 42.9%, *P* < 0.001), greater mechanical ventilation rates (92.5% vs. 65.1%, *P* < 0.001), and greater rates of lactate ≥2.6 mmol/L (58.8% vs. 28.2%, *P* < 0.001).

**Table 5:** Characteristics of patients in the low and high fluid intake groups within the first 24 h.

Variables	Fluid intake within the first 24 h (ml.kg^−1^)		*P* value
	<147 (*n* = 522)	≥147 (*n* = 522)	
Fluid intake (ml.kg^−1^)	66.4 (40.2, 101.5)	243.2 (191.2, 344.1)	<0.001
Duration of ICU stay (hours)	83.6 (50.9, 183.1)	118.2 (63.8, 244.2)	<0.001
CRRT—*n* (%)			<0.001
Yes	43 (8.2%)	82 (15.7%)	
No	479 (91.8%)	440 (84.3%)	
Vasopressors—*n* (%)			<0.001
Yes	224 (42.9%)	440 (84.3%)	
No	298 (57.1%)	82 (15.7%)	
Ventilation—*n* (%)			<0.001
Yes	340 (65.1%)	483 (92.5%)	
No	182 (34.9%)	39 (7.5%)	
Lactate (mmol/L)—*n* (%)			<0.001
<2.6	244 (46.7%)	201 (38.5%)	
≥2.6	147 (28.2%)	307 (58.8%)	
No test	131 (25.1%)	14 (2.7%)	

CRRT, continuous renal replacement therapy; ICU, intensive care unit.

**Figure 6:**
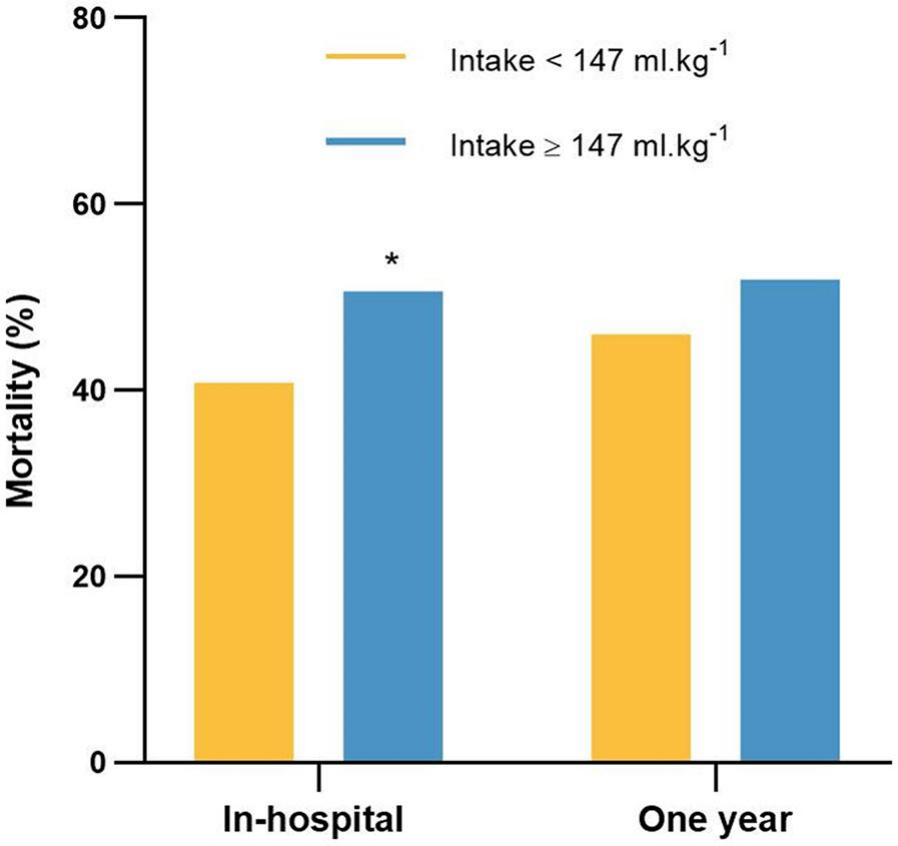
The in-hospital mortality and 1-year mortality for patients in the low-fluid intake and high-fluid intake groups. *P < 0.05, vs. intake <147 ml.kg^−1^.

The in-hospital mortality was 50.6% (264 deaths of 522 patients) in the high-fluid intake group, which was significantly greater than the 40.8% (213 deaths of 522 patients) in the low-fluid intake group (*P* = 0.002; Figure 6). The 1-year mortality was 51.9% (271 deaths of 522 patients) in the high-fluid intake group and 46% (240 deaths of 522 patients) in the low-fluid intake group. Moreover, there was no significant difference between the two groups (*P* = 0.055).

The associations between fluid intake within 24 h after admission to the ICU and in-hospital mortality in CA patients are shown in Table 6. After adjusting for the effects of confounding factors, the high-fluid intake group was not associated with increased in-hospital mortality (OR 0.841, 95% CI 0.587–1.204; *P* = 0.344) compared with the low-fluid intake group.

**Table 6:** Logistic regression analysis of the association of fluid intake within the first 24 h with in-hospital mortality.

Fluid intake (ml.kg^−1^)	Non-adjusted			Adjusted^a^		
	OR	95% CI	*P* value	OR	95% CI	*P* value
<147	Reference	-	-	Reference	-	-
≥147	1.484	1.162–1.896	0.002	0.841	0.587–1.204	0.344

OR, odd ratio; CI, confidence interval.

a Variables included in the model, age, gender, ethnicity, SBP, HR, RR, SpO_2_, SOFA, SAPSⅡ, GCS, vasopressor use, mechanical ventilation ratios, diuretics use, CRRT, AHF, CHF, coronary heart disease, blood creatinine, BUN, glucose, sodium, calcium, pH, lactate, fluid intake during the first 24 h, length of stay in ICU.

## Discussion

This study aims to better investigate the effects of postresuscitation continuous fluid balance and early fluid intake in CA patients through a retrospective study of the MIMIC-IV database to simultaneously evaluate the associations between the mean daily fluid balance within 7 days and fluid intake within 24 h after admission to the ICU and mortality for hospitalization and 1-year in CA patients. The results showed that mean daily fluid balance ≥38 ml.kg^−1^ within 7 days after admission to the ICU was associated with increased in-hospital mortality and 1-year mortality in CA patients. In addition, fluid intake ≥147 ml.kg^−1^ within 24 h was not associated with increased in-hospital mortality.

IVF therapy, as a basic therapy in critical care, may increase cardiac output and improve organ perfusion. Frank–Starling's law suggests that an increase in ventricular end-diastolic volume within a certain range can enhance ventricular contractility, but after the preload reaches the optimal volume, further increases in preload at this time may lead to myocardial injury (9). There is substantial evidence indicating that an aggressive fluid resuscitation strategy is harmful. It can cause pulmonary edema, acute respiratory distress syndrome, and blood loss, trigger dilutional coagulopathy, exacerbate tissue hypoxia, thereby increasing the risk of metabolic acidosis (14–18). The present results also showed that mean daily fluid balance ≥38 ml.kg^−1^ within 7 days after admission to the ICU was associated with increased in-hospital mortality and 1-year mortality in CA patients. In addition, nonsurvivors had higher daily fluid intake and lower output, which resulted in a greater daily fluid balance than that of survivors. These results suggest that excessive fluid intake has a deleterious effect on the prognosis of CA patients. Therefore, it is crucial to pay close attention to the changes in fluid requirements at different stages of the resuscitation process to improve the prognosis of CA patients.

Although a high fluid intake within 24 h was not associated with in-hospital mortality, the use of vasopressors, the use of mechanical ventilation, and the use of CRRT were greater, and hospital stays were longer in the high-fluid intake group. Deaths within the first 24 h after ROSC typically result from refractory shock producing recurrent CA or persistent hemodynamic instability leading to multiorgan system failure (19–22). CA patients appear to have a higher inherent risk of myocardial dysfunction which can be brought about by myocardial stunning after cardiac arrest (23), which may influence the ability to handle volume challenges in the post-cardiac arrest state. However, the database lacked a record of the patient's postresuscitation hemodynamic data, such as time to CA, the presence or absence of spontaneous circulatory recovery, cardiac output, central venous pressure and indicators to guide IVF, and echocardiographic monitoring of cardiac function. Therefore, we could not determine whether these factors contributed to the fact that a high fluid intake within 24 h was not associated with an increased mortality in CA patients. A study conducted by Fahad Gul showed that there was no association between the volume of resuscitated fluid within 24 h (<30 vs. >30 ml.kg^−1^) and in-hospital mortality in CA patients (12). They also found that fluid resuscitation was independently associated with greater use of vasopressors and duration of mechanical ventilation, which is consistent with our findings. However, the optimal fluid volume for fluid resuscitation within 24 h after CA remains uncertain, and early fluid resuscitation after CA may be influenced by a variety of factors. Typically, an infusion response strategy (which is defined as a 10%–15% increase in a patient's cardiac output) is recommended as the gold standard for guiding IVF after CA (24). Because the effects of IVF on cardiac output are transient, whereas the effects on central venous pressure and venous congestion are more persistent, a resuscitation strategy of multiple IVFs based only on the response to cardiac output may result in multiorgan damage. As fluid tolerance strategies are proposed, by examining the potential benefits or harms associated with additional fluid management. Unlike fluid responsiveness, fluid tolerance is not based on a single measure, but rather assesses a holistic approach by examining the patient's history, current clinical presentation, bedside ultrasound, and measurements of capillary integrity and extravascular fluid accumulation to determine whether the patient is more likely to benefit from IVF (25). In summary, the deleterious effects of excessive fluid intake need to be evaluated, and IVF, which is used only as a bridge to resuscitation, is intended to maintain circulatory stability in CA patients until the potential etiology of CA is controlled.

Postresuscitation shock occurs in 50%–70% of CA patients, and it is an early and transient complication of the postresuscitation (26). The pathophysiology of postresuscitation shock is complex and is caused mainly by myocardial dysfunction, vascular paralysis and hypovolemia (27). Hypovolemia can lead to vasoconstriction and inadequate perfusion, reducing oxygen delivery to organs and peripheral tissues and leading to organ dysfunction. Hypovolemia after CA is common but is often undertreated because physicians are concerned about fluid overload when performing fluid resuscitation on patients with underlying myocardial dysfunction. Ultimately, there is an increased chance of a vicious cycle of postresuscitation shock, including myocardial injury, reduced cardiac output, triggering splanchnic injury leading to endotoxin release, and vasopressor resistance; requiring higher doses of vasoactive drugs; further exacerbating myocardial injury and organ failure; and increasing mortality (22, 26). A retrospective study suggests that early exposure to arterial hypotension after ROSC was an independent predictor of death (28).

Patients who are hemodynamically unstable after ROSC are often administered with vasopressors to maintain arterial pressure, and thus ameliorate tissue hypoperfusion and arterial hypotension (29–31). It has been shown that vasopressor therapy should be initiated in patients with severe hypotension at the same time as infusion, as the degree and duration of hypotension are associated with increased mortality in patients discharged from the hospital (32). However, one study revealed that early post-ROSC hemodynamic resuscitation resulting in a higher MAP (≥65 mmHg) when fluid is used preferentially rather than vasopressors is associated with improved survival to hospital discharge (33). Therefore, the effect of vasopressors on CA patients in the early post-ROSC period has not been determined. At present, there are limited data on the prognostic impact of prioritizing fluid resuscitation or prioritizing the use of vasopressors to maintain MAP stabilization in the management of patients with post-CA shock, and the early use of vasopressors may prevent excessive fluid resuscitation. However, early use of vasopressors may increase venous tone and limit the amount of fluid required during initial resuscitation, whereas cardiac output and organ oxygen delivery can be increased only if venous return is increased by adequate IVF. The present study evaluated the use of five vasopressors within 24 h of admission to the ICU and revealed that the use of vasopressors was greater in the high-fluid intake group and nonsurvivors. Multivariate logistic regression analysis also revealed that vasopressors were an independent risk factor for in-hospital mortality and 1-year mortality in CA patients. Therefore, vasopressors should be used cautiously with monitoring of the circulation and adequate assessment of the patient's fluid tolerance.

The composition of each intravenous solution may affect organ function and patient prognosis (34, 35). For adult patients in the emergency department and ICU, compared to saline, balanced crystals reduce catecholamine use, death and renal dysfunction (36, 37). Moreover, albumin increases mortality in traumatic brain injury (38) but plays a beneficial role in the treatment of infectious shock (39). Semisynthetic colloids increase the risk of acute kidney injury and should not be used for fluid resuscitation in most critically ill patients (40). Practical, evidence-based fluid resuscitation recommendations encourage the use of isotonic buffered salt solution as a first-line resuscitation fluid (35). The type of fluid and the amount of IVF that may impair acid‒base balance (41). According to Stewart's theory, hydroxyethyl starch and dextrose solution will exhibit acidotic effects. Ringer's lactate and plasmalytes easily cause alkalosis (42). In addition, inadequate tissue oxygenation after CA results in the conversion of cellular tissue oxygenation from aerobic to anaerobic metabolism, causing an increase in lactate (8). This study also demonstrated that patients in the high fluid intake group and the >79 ml.kg^−1^ group had a higher rate of lactate ≥2.6 mmol/L. Furthermore, a high lactate concentration (≥2.6 mmol/L) was an independent risk factor for in-hospital mortality and 1-year mortality in CA patients. Therefore, the circulatory status of the patient should be adequately assessed prior to IVF therapy, considering the benefits and potential risks of the type and volume of IVF, and selecting the appropriate IVF solution. Regrettably, this study did not examine the effects of different fluid types on acid-base balance, and further research will be conducted in the future to investigate the beneficial and harmful effects of different fluid compositions on CA patients.

Different etiologies may lead to different volume states in patients. In the present study, nonsurvivors had higher daily fluid intake and lower output, which resulted in a greater daily fluid balance than that of survivors. This could be because nonsurvivors are hemodynamically unstable and require more IVF to maintain arterial blood pressure. However, the lower fluid intake in survivors could be because they had a greater incidence of AHF, CHF, hypertension, CHD and COPD than did nonsurvivors, and these diseases limited IVF in survivors. Therefore, the present study included coexisting diseases as well as electrolyte disorders that predispose patients to CA, including hypertension, CHD, diabetes mellitus, AHF, CHF, COPD, and blood potassium, sodium, and calcium levels. In a baseline comparison of the four mean daily fluid balance groups, the prevalence of AHF, CHF and CHD, and blood calcium were lower in the >79 ml.kg^−1^ group than in the <14 ml.kg^−1^ group, whereas there were no significant differences in the other variables. This suggests that patients in the >79 ml.kg^−1^ group had a better pre-CA functional status than those in the <14 ml.kg^−1^ group and that IVF may not be limited by cardiac causes in the >79 ml.kg^−1^ group, whereas patients in the >79 ml.kg^−1^ group had a higher in-hospital mortality and 1-year mortality than those in the <14 ml.kg^−1^ group, and only CHD was associated with mortality in the multivariate logistic regression models. Thus, the results of this study suggest that the cause of CA has less effect on the prognosis of patients.

In this study, we also found that patients in the >79 ml.kg^−1^ group had higher SOFA scores and SAPS II scores, lower GCS scores compared with the other three groups. Moreover, in the multivariate logistic regression analysis of in-hospital mortality, it showed that SOFA score and GCS score were associated with in-hospital mortality, indicating that patients in the high fluid balance group had worse circulatory, respiratory, hepatic, renal functions and neurologic functions than those in the low fluid balance group, which led to poorer tolerance of aggressive fluid resuscitation for 7 consecutive days, and ultimately led to an increase in in-hospital mortality and 1-year mortality for patients in the high fluid balance group.

Current critical care and cardiology social guidelines do not provide guidance on the optimal dose of IVF for use during resuscitation after CA, and the literature on whether to administer IVF after resuscitation from CA is limited due to the lack of high-quality trials (43). There are significant differences in IVF therapy for CPR in current clinical work. IVF therapy plays a crucial role in establishing and maintaining cellular homeostasis in hospitalized patients. IVF therapy is one of the most common treatments used in hospitals as a basic treatment for critically ill patients. Vincent and De Backer (44) divide the process of resuscitation of critically ill patients into four distinct phases: resuscitation, optimization, stabilization, and de-escalation. It clearly indicates immediate fluid management for life-threatening conditions associated with impaired tissue perfusion during the resuscitation phase. In the optimization phase, the focus of fluid therapy shifts from saving the patient's life to ensuring that sufficient blood and oxygen are delivered to the organs. The purpose of this phase is to prevent subsequent organ dysfunction and failure due to inadequate perfusion and tissue edema. The stabilization phase reflects the patient being in a stable state and fluid therapy is now used only for the continued maintenance of normal fluid loss. The de-escalation phase minimizes IVF and mobilizes additional fluids to optimize fluid balance (35, 45, 46). Fluid therapy to improve microvascular blood flow and increase cardiac output is an important part of the treatment of any form of shock. Fluid resuscitation after CA is a dynamic process that depends on the patient's condition, and physicians need to evaluate the volume and type of IVF not only at the beginning but also during subsequent days. Thus, it is more meaningful to study a single time period than a few time points. There was a lack of unified time points in previous studies (11, 47). The present study has certain advantages over other previous studies. In the present study, we evaluated not only the early infusion of CA (24 h after admission to the ICU) but also the fluid balance within the first 7 days after admission to the ICU. This was the first study to analyze the association between fluid balance for 7 days and in-hospital and 1-year mortality in CA patients. Our study can provide insight into current management patterns after CA and provide a database for future studies of resuscitation after CA.

There were several limitations in our study. First, this study investigated only the association between early fluid intake and 7-day mean fluid balance and mortality in CA patients, but key questions regarding the optimal fluid composition have not been answered. Second, there was high heterogeneity among the critical patients. Although multivariate logistic regression analysis was used to adjust for the effects of confounding factors, there may still be some unknown factors that interfere with the observed results. Third, the database lacked a record of the patient's postresuscitation hemodynamic data, such as the time of cardiac arrest, the presence of return of spontaneous circulation and indicators to guide IVF and the absence of hemodynamic data (such as cardiac output and central venous pressure) limits mechanistic insights. Fourth, this was an observational study conducted at a single center, limiting the generalizability of the results to other centers; external validation from other medical institutions is needed. Fifth, this study found that mean daily fluid balance ≥38 ml.kg^−1^ within 7 days after admission to the ICU was associated with increased in-hospital mortality and 1-year mortality in CA patients. The findings represent associations, not causation. During fluid resuscitation, the patient's circulation needs to be closely monitored, and the integration of dynamic assessments (such as ultrasound and hemodynamic monitoring) for individualized fluid management therapy is critical for improving patient prognosis. In future prospective studies, it is necessary to determine the optimal fluid type for fluid resuscitation, and explore the impacts of saline and colloid solutions on acid-base balance and patients' prognosis. Additionally, individualized fluid resuscitation protocols should be formulated for different complications of patients with CA, and the use of vasoactive drugs should be standardized to provide precise guidance for clinical resuscitation.

## Conclusion

Mean daily fluid balance ≥38 ml.kg^−1^ within 7 days after admission to the ICU was associated with increased in-hospital mortality and 1-year mortality in cardiac arrest patients. Fluid intake ≥147 ml.kg^−1^ within 24 h was not associated with increased in-hospital mortality. This study may provide guidance for current post-cardiac arrest management and theoretical support for future research on post-cardiac arrest resuscitation.

## Data Availability

The original contributions presented in the study are included in the article/Supplementary Material, further inquiries can be directed to the corresponding author.
