# Correction: Effect of fluid balance situation within 7 days and early fluid intake after admission to the intensive care unit on in-hospital mortality and 1-year mortality in patients with cardiac arrest: a retrospective study from the MIMIC IV database

**DOI:** 10.3389/fcvm.2025.1752768

**Published:** 2026-01-02

**Authors:** Lei Zhang, Chang Liu, Xin Sui, Jian Zhang, Wenjia Xu, Yufei Sun, Chengke Yin, Fei Han

**Affiliations:** Department of Anesthesiology, Harbin Medical University Cancer Hospital, Harbin, Heilongjiang, China

**Keywords:** cardiac arrest, fluid resuscitation, fluid balance, intravenous fluid, medical information mart for intensive care IV

Author Lei Zhang was erroneously assigned to affiliation “Department of Anesthesiology, The Second Affiliated Hospital of Kunming Medical University, Kunming, Yunnan, China.” This affiliation has now been removed for author Lei Zhang.

Affiliation “Department of Anesthesiology, Harbin Medical University Cancer Hospital, Harbin, Heilongjiang, China” was unassigned for author Lei Zhang. This affiliation has now been assigned for author Lei Zhang.

The original version of this article has been updated.

