# Supplementary material for: Effect of fluid balance situation within 7 days and early fluid intake after admission to the intensive care unit on in-hospital mortality and 1-year mortality in patients with cardiac arrest: a retrospective study from the MIMIC IV database
Source: Front Cardiovasc Med. 2025 Nov 11;12:1519306. doi: 10.3389/fcvm.2025.1519306 (PMC12644014; doi:10.3389/fcvm.2025.1519306)
Supplement: Supplementary file 1 [file Table1.docx]

**Supplementary Table 1.** Details of missing values.

| Variables | The number of missing values | The percent of missing values |
| --- | --- | --- |
| GCS | 2 | 0.19% |
| SBP | 2 | 0.19% |
| DBP | 2 | 0.19% |
| SpO_2_ | 4 | 0.38% |
| Glucose | 1 | 0.10% |
| Calcium | 45 | 4.30% |
| Sodium | 1 | 0.10% |
| Potassium | 2 | 0.19% |
| Lactate | 145 | 13.90% |
| pH | 122 | 11.70% |

SBP, systolic blood pressure; DBP, diastolic blood pressure; SpO_2_, arterial oxyhemoglobin saturation.
