# Supplementary material for: Effect of fluid balance situation within 7 days and early fluid intake after admission to the intensive care unit on in-hospital mortality and 1-year mortality in patients with cardiac arrest: a retrospective study from the MIMIC IV database
Source: Front Cardiovasc Med. 2025 Nov 11;12:1519306. doi: 10.3389/fcvm.2025.1519306 (PMC12644014; doi:10.3389/fcvm.2025.1519306)
Supplement: Supplementary file 2 [file Table2.docx]

**Supplementary Table 2.** Univariate logistic regression analysis of in-hospital mortality.

| Variables | Univariate model | | |
| --- | --- | --- | --- |
|  | OR | 95% CI | *P* value |
| Age | 1.011 | 1.004 - 1.019 | 0.004 |
| Male | 0.660 | 0.514 - 0.847 | 0.001 |
| Ethnicity |  |  | 0.011 |
| White | Reference |  |  |
| Black | 1.249 | 0.831 - 1.875 | 0.285 |
| Other | 1.531 | 1.157 - 2.025 | 0.003 |
| Weight | 0.996 | 0.990 - 1.002 | 0.163 |
| MAP | 0.991 | 0.979 - 1.002 | 0.108 |
| SBP | 0.991 | 0.983 - 0.999 | 0.025 |
| DBP | 0.993 | 0.982 - 1.004 | 0.223 |
| HR | 1.017 | 1.010 - 1.024 | <0.001 |
| RR | 1.092 | 1.059 - 1.126 | <0.001 |
| SpO_2_ | 0.929 | 0.886 - 0.975 | 0.003 |
| Scoring systems |  |  |  |
| SOFA | 1.111 | 1.079 - 1.143 | <0.001 |
| SAPSII | 1.037 | 1.028 - 1.045 | <0.001 |
| GCS | 0.912 | 0.888 - 0.936 | <0.001 |
| Treatment |  |  |  |
| Vasopressor | 2.181 | 1.679 - 2.834 | <0.001 |
| Ventilation | 1.491 | 1.100 - 2.020 | 0.010 |
| Diuretics | 0.497 | 0.383 - 0.646 | <0.001 |
| CRRT | 1.724 | 1.182 - 2.516 | 0.005 |
| Comorbidities |  |  |  |
| AHF | 0.688 | 0.505 - 0.937 | 0.018 |
| CHF | 0.741 | 0.577 - 0.952 | 0.019 |
| Hypertension | 0.807 | 0.630 - 1.034 | 0.090 |
| Diabetes mellitus | 1.196 | 0.884 - 1.616 | 0.245 |
| CHD | 0.550 | 0.428 - 0.707 | <0.001 |
| COPD | 0.491 | 0.240 - 1.005 | 0.052 |
| Laboratory tests |  |  |  |
| Creatinine | 1.136 | 1.044 - 1.236 | 0.003 |
| BUN | 1.019 | 1.013 - 1.025 | <0.001 |
| Glucose | 1.004 | 1.002 - 1.006 | <0.001 |
| Sodium | 1.032 | 1.005 - 1.059 | 0.019 |
| Potassium | 0.970 | 0.784 - 1.201 | 0.779 |
| Calcium | 0.847 | 0.729 - 0.984 | 0.030 |
| pH |  |  | <0.001 |
| <7.35 | Reference |  |  |
| ≥7.35 | 0.557 | 0.429 - 0.724 | <0.001 |
| No test | 0.523 | 0.348 - 0.787 | <0.001 |
| Lactate |  |  | <0.001 |
| <2.6 mM | Reference |  |  |
| ≥2.6 mM | 2.826 | 2.155 - 3.706 | 0.001 |
| No test | 1.099 | 0.744 - 1.625 | 0.634 |
| Mean daily fluid balance(ml.kg^-1^) |  |  | <0.001 |
| <14 | Reference |  |  |
| 14-37 | 1.841 | 1.279 - 2.650 | 0.001 |
| 38-79 | 3.062 | 2.130 - 4.403 | <0.001 |
| >79 | 3.415 | 2.372 - 4.915 | <0.001 |
| Length of stay in ICU | 0.999 | 0.998 - 1.000 | 0.005 |

OR: odd Ratio; CI: Confidence Interval.

MAP, mean arterial pressure; SBP, systolic blood pressure; DBP, diastolic blood pressure; HR, heart rate; RR, respiration rate; SpO_2_, arterial oxyhemoglobin saturation; SOFA, sequential organ failure assessment; SAPSII, simplified acute physiology scores II; GCS, glasgow coma scale; CRRT, continuous renal replacement therapy; AHF, acute heart failure; CHF, congestive heart failure; CHD, coronary heart disease; COPD, chronic obstructive pulmonary disease; BUN, blood urea nitrogen; ICU, intensive care unit.
