# Supplementary material for: Effect of fluid balance situation within 7 days and early fluid intake after admission to the intensive care unit on in-hospital mortality and 1-year mortality in patients with cardiac arrest: a retrospective study from the MIMIC IV database
Source: Front Cardiovasc Med. 2025 Nov 11;12:1519306. doi: 10.3389/fcvm.2025.1519306 (PMC12644014; doi:10.3389/fcvm.2025.1519306)
Supplement: Supplementary file 3 [file Table3.docx]

**Supplementary Table 3.** Univariate logistic regression analysis of one-year mortality.

| Variables | Univariate model | | |
| --- | --- | --- | --- |
|  | OR | 95% CI | P value |
| Age | 1.013 | 1.005 - 1.021 | 0.001 |
| Male | 0.698 | 0.544 - 0.896 | 0.005 |
| Ethnicity |  |  | 0.114 |
| White | Reference |  |  |
| Black | 1.170 | 0.780 - 1.756 | 0.448 |
| Other | 1.341 | 1.014 - 1.772 | 0.040 |
| Weight | 0.996 | 0.990 - 1.002 | 0.156 |
| MAP | 0.990 | 0.979 - 1.001 | 0.086 |
| SBP | 0.991 | 0.983 - 0.999 | 0.033 |
| DBP | 0.994 | 0.983 - 1.005 | 0.271 |
| HR | 1.018 | 1.011 - 1.025 | <0.001 |
| RR | 1.073 | 1.041 - 1.106 | <0.001 |
| SpO_2_ | 0.938 | 0.895 - 0.984 | 0.009 |
| Scoring systems |  |  |  |
| SOFA | 1.110 | 1.078 - 1.142 | <0.001 |
| SAPSII | 1.035 | 1.027 - 1.044 | <0.001 |
| GCS | 0.923 | 0.899 - 0.947 | <0.001 |
| Treatment |  |  |  |
| Vasopressor | 2.171 | 1.676 - 2.811 | <0.001 |
| Ventilation | 1.489 | 1.102 - 2.011 | 0.009 |
| Diuretics | 0.546 | 0.420 - 0.579 | <0.001 |
| CRRT | 1.793 | 1.222 - 2.629 | 0.003 |
| Comorbidities |  |  |  |
| AHF | 0.746 | 0.550 - 1.011 | 0.059 |
| CHF | 0.851 | 0.664 - 1.090 | 0.202 |
| Hypertension | 0.731 | 0.572 - 0.936 | 0.013 |
| Diabetes mellitus | 1.115 | 0.827 - 1.504 | 0.474 |
| CHD | 0.608 | 0.474 - 0.779 | <0.001 |
| COPD | 0.625 | 0.318 - 1.228 | 0.172 |
| Laboratory tests |  |  |  |
| Creatinine | 1.185 | 1.085 - 1.294 | <0.001 |
| BUN | 1.021 | 1.015 - 1.028 | <0.001 |
| Glucose | 1.003 | 1.002 - 1.005 | <0.001 |
| Sodium | 1.026 | 1.000 - 1.053 | 0.051 |
| Potassium | 1.078 | 0.871 - 1.333 | 0.491 |
| Calcium | 0.863 | 0.744 - 1.001 | 0.052 |
| pH |  |  | <0.001 |
| <7.35 | Reference |  |  |
| ≥7.35 | 0.591 | 0.455 - 0.767 | <0.001 |
| No test | 0.606 | 0.406 - 0.904 | 0.014 |
| Lactate |  |  | <0.001 |
| <2.6 mM | Reference |  |  |
| ≥2.6 mM | 2.678 | 2.045 - 3.506 | <0.001 |
| No test | 1.153 | 0.787 - 1.689 | 0.466 |
| Mean daily fluid balance(ml.kg^-1^) |  |  | <0.001 |
| <14 | Reference |  |  |
| 14-37 | 1.878 | 1.361 - 2.680 | 0.001 |
| 38-79 | 2.893 | 2.023 - 4.136 | <0.001 |
| >79 | 2.985 | 2.087 - 4.270 | <0.001 |
| Length of stay in ICU | 0.999 | 0.998 - 1.000 | 0.006 |

OR: odd Ratio; CI: Confidence Interval.

MAP, mean arterial pressure; SBP, systolic blood pressure; DBP, diastolic blood pressure; HR, heart rate; RR, respiration rate; SpO_2_, arterial oxyhemoglobin saturation; SOFA, sequential organ failure assessment; SAPSII, simplified acute physiology scores II; GCS, glasgow coma scale; CRRT, continuous renal replacement therapy; AHF, acute heart failure; CHF, congestive heart failure; CHD, coronary heart disease; COPD, chronic obstructive pulmonary disease; BUN, blood urea nitrogen; ICU, intensive care unit.
